# Discrimination reported by people with schizophrenia: cross-national variations in relation to the Human Development Index

**DOI:** 10.1017/S2045796023000781

**Published:** 2023-11-21

**Authors:** P. C. Gronholm, S. Ali, E. Brohan, G. Thornicroft

**Affiliations:** Health Service and Population Research Department, Institute of Psychiatry, Psychology & Neuroscience, King’s College London, London, UK

**Keywords:** cultural variation, discrimination, mental illness stigma, multicultural, schizophrenia, severe mental illness, social distance

## Abstract

**Aims:**

Mental health related stigma and discrimination is a universal phenomenon and a contributor to the adversity experienced by people with schizophrenia. Research has produced inconsistent findings on how discrimination differs across settings and the contextual factors that underpin these differences. This study investigates the association between country-level Human Development Index (HDI) and experienced and anticipated discrimination reported by people with schizophrenia.

**Methods:**

This study is a secondary data analysis of a global cross-sectional survey completed by people living with schizophrenia across 29 countries, between 2005 and 2008. Experienced and anticipated discrimination were assessed using the Discrimination and Stigma Scale (DISC-10). Countries were classified according to their 2006 HDI. Negative binomial and Poisson regression analyses with a robust standard errors approach were conducted to investigate associations between country-level HDI and discrimination.

**Results:**

In the regression analyses, no evidence was found for a linear association between HDI and experienced or anticipated discrimination. Further exploratory analyses showed a significant non-linear association between HDI ratings and experienced discrimination. Participants in “high” and “very high” HDI countries reported more experienced discrimination compared to those in “medium” HDI countries.

**Conclusions:**

HDI does, to some extent, appear to be associated with how far discrimination is experienced across different contexts. More high-quality cross-national research, including research focused on “medium” and “low” countries, is needed to substantiate these findings and identify underlying factors that may explain the pattern observed for experienced discrimination, including generating new datasets that would enable for these analyses to be repeated and contrasted with more recent data. An in-depth understanding of these factors will further aid the adaptation of cross-cultural and context specific anti-stigma interventions in future.

## Introduction

Schizophrenia is a severe mental health condition affecting approximately 21 million people globally, accounting for a substantial proportion of the global mental health burden worldwide (Charlson *et al.*, 2018; He *et al.*, 2020). Schizophrenia can be associated with a series of adverse social, health and economic consequences, such as premature mortality; higher incidence of comorbid disease; poverty and homelessness; increased direct costs to the health and social sectors; and indirect economic impacts secondary to productivity losses from unemployment and relatively high rates of institutionalisation and incarceration (Thornicroft *et al.*, 2022). Mental health-related stigma and discrimination is another adverse experience which can have important adverse impacts for many people with schizophrenia (Charlson *et al.*, 2018; He *et al.*, 2020). Indeed, schizophrenia is reported to be the most stigmatised psychiatric diagnosis (Angermeyer and Matschinger, 2003; Ben-Zeev *et al.*, 2010; Corrigan, 2007; Dinos *et al.*, 2004; Hazell *et al.*, 2022; Pescosolido *et al.*, 2010; Phelan *et al.*, 2000), and compared to other diagnoses and/or mental health conditions people with schizophrenia are considered to be more unpredictable and dangerous, with reduced prospects of recovery (Durand-Zaleski *et al.*, 2012; Neal, 2021; Wood *et al.*, 2014). It has also been reported that people diagnosed with schizophrenia were more likely than other groups to experience discrimination, such as verbal and physical abuse and social ostracizing (Dinos *et al.*, 2004), and that even psychosis risk elicits more social distance than other diagnoses (Yang *et al.*, 2013).

Discrimination can be broadly categorised into two main subtypes; experienced and anticipated discrimination (Brohan *et al.*, 2013). The former reflects how people are disadvantaged by others’ discriminatory behaviour, whereas the latter refers to a persons’ anticipatory behaviour because of an expectation that discrimination will occur. Discrimination can represent a significant life barrier in gaining employment, accessing education and healthcare services, securing housing or forming interpersonal relationships (Thornicroft *et al.*, 2022). Indeed, for many people, discrimination is described as worse than the mental health condition itself (Thornicroft, 2006; Thornicroft *et al.*, 2016, 2022).

Stigma and discrimination are found universally, but there are also cultural and contextual variations in how they are manifested and experienced and their consequences. These differences can be understood through considering the role of culture in how mental illnesses are conceptualised, other sociocultural factors and the social structure of different communities (Semrau *et al.*, 2015). For example, it has been suggested that stigma is less pronounced in cultures with collectivist values and strong family cohesiveness, which can act as protective barriers to alienation and social exclusion (Lasalvia *et al.*, 2015; Papadopoulos *et al.*, 2013; Semrau *et al.*, 2015). Socioeconomic factors may also play a pivotal role in how stigma is experienced and the weight of its harmful effects on individuals (Semrau *et al.*, 2015; Yang *et al.*, 2014). In low resource settings, financial insecurity as a result of illness, combined with a lack of universal health coverage, can pose a severe threat to individuals and their families (Ebuenyi *et al.*, 2019; Koschorke *et al.*, 2014), and this may be a driver of discrimination. It can also be argued that loss of employment and financial insecurity in high-income settings (HICs), which place great emphasis on autonomy and self-sufficiency, could result in more social exclusion and discrimination (Papadopoulos *et al.*, 2013). Generally, research conducted in different regions is difficult to compare due to varying methodologies and measurement tools. To remedy this methodological shortcoming, the INDIGO (International Study of Discrimination and Stigma Outcomes) (Thornicroft *et al.*, 2019) programme has assessed discrimination using a standardised scale across multiple countries. One key study within this programme investigated discrimination among people with a diagnosis of schizophrenia (Thornicroft *et al.*, 2009). These data have also been considered specifically in view of discrimination related to medical services (Harangozo *et al.*, 2013), and anticipated discrimination in particular (Üçok *et al.*, 2012). A parallel INDIGO study (Lasalvia *et al.*, 2015) investigated experienced and anticipated discrimination among people with major depressive disorder in relation to individual level and country-level factors, including the international standardised measure, Human Development Index (HDI). A comparable exploration has not been conducted with the INDIGO-Schizophrenia data.

The HDI is a useful index to assess the overall “richness of human life” (United Nations Development Programme, n.d.) and offers a homogenous way of measuring contextual differences across regions (Khawas, 2016). Building on this work, this study aims to investigate whether country-level HDI is associated with individual-level experienced and anticipated discrimination in a cross-sectional international survey of people with schizophrenia.

## Methods

### Design

Data were collected between 2005 and 2008 through a cross-sectional survey as part of the INDIGO-Schizophrenia study. Full details of study are provided elsewhere (Thornicroft *et al.*, 2009). In brief, it explored variations in experiences of discrimination considering data from 29 countries (Austria, Belgium, Brazil, Bulgaria, Canada, Cyprus, Finland, France, Germany, Greece, India, Italy, Japan, Lithuania, Malaysia, Netherlands, Norway, Poland, Portugal, Romania, Serbia, Slovakia, Slovenia, Spain, Switzerland, Tajikistan, Turkey, United Kingdom and United States of America). These data were originally collected to explore the global pattern of discrimination; this study reports on secondary analyses of these data.

### Participants

All participants were elected by site directors and recruited from local psychiatric services. Each site had a target to recruit 25 participants; samples ranged from 18 to 50 participants per site (mean 28/mode 25 participants). To optimise feasibility, a convenience sampling strategy with no formal sample size calculations was employed. In each site, teams were asked to identify individuals whom they believed, collectively, fairly represented the broader population of individuals receiving treatment for schizophrenia within their respective local psychiatric services. This encompassed individuals receiving care in various settings, including inpatient, day-patient, outpatient, and community-based services. The inclusion criteria were: (i) confirmed clinician diagnosis of schizophrenia; (ii) undergoing treatment from a psychiatric service (i.e. including but not limited to community, inpatient, day setting or outpatient facilities); (iii) ability to provide written informed consent; (iv) fluency in the local language; and (v) aged 18 years or above. Acutely unwell individuals were not eligible to participate in the study.

### Ethical approval

Written informed consent was obtained from all participants. The authors assert that all procedures contributing to this work comply with the ethical standards of the relevant national and institutional committees on human experimentation and with the Helsinki Declaration of 1975, as revised in 2008. The London School of Hygiene and Tropical Medicine (LSHTM) MSc Ethics Committee confirmed ethical approval of this study (reference: 21736). This study reports on secondary analyses from the original study data collection, for which Ethical approval was obtained from the KCL Research Ethics Committee (reference 039/04) including permission to use anonymised data for secondary analyses. Local ethical approval for the primary study was granted by review board situated at each study site.

### Measures

#### Primary exposure measure: Human Development Index (HDI)

All 29 countries were rated according to their 2006 HDI, corresponding to the approximate year of data collection. The HDI reflects a composite measure to assess a country’s average achievement across key development indices (health and longevity of life; education level; and satisfactory standard of living) (Khawas, 2016). Scores range from 0 to 1; higher scores denote higher level of human development. The HDI can also be grouped into categorical ratings: “medium”; “high”; and “very high” using the United Nations Development Programme (UNDP) threshold values (≥ 0.444 = low HDI, ≥ 0.544 = medium HDI, ≥ 0.675 = high HDI, ≥ 0.851 = very high HDI) (United Nations Development Programme, 2018).

#### Primary outcome measure: Discrimination and Stigma Scale (DISC-10)

The DISC-10 is a 36-item measure assessing the nature, direction and severity of discrimination from the perspective of people living with a mental illness (Thornicroft *et al.*, 2009). This study considered items assessing experienced discrimination and anticipated discrimination. The scale was later further developed into the DISC-12 version (Brohan *et al.*, 2013), and the shorter DISCUS version (Bakolis *et al.*, 2019; Brohan *et al.*, 2022).

Experienced discrimination was assessed via 32 items measuring participants’ experiences of differential treatment (either to their advantage or disadvantage; the latter representing experienced negative discrimination) across a number of life domains (e.g. interpersonal relationships; housing; education; employment; travel; interaction with health and social services). Responses were scored on a 7-point Likert scale (ranging from −3 = “strong disadvantage” to + 3 = “strong advantage”). An “experienced discrimination” score was formulated through summating negative (−1 = slight disadvantage, −2 = moderate disadvantage, −3 = strong disadvantage) scale-point responses.

Anticipated discrimination was assessed via four items measuring the degree to which participants limit their participation in aspects of daily life (e.g. employment, education, leisure activities, interpersonal relationships). Responses were scored on a 3-point Likert scale (0 = not at all, 1 = a little, 2 = a lot). An “anticipated discrimination” score was formulated through summating positive (above 0) scale-point responses.

The DISC-10 items were forward and back translated using focus group discussions involving 6–10 people with schizophrenia in each study site, to ensure local language versions of the measure were understandable and contextually adapted (Knudsen *et al.*, 2000; Rose *et al.*, 2011; Thornicroft *et al.*, 2009).

#### Socio-demographic and clinically related variables

Data were collected on participant age (continuous variable), number of years of education (continuous); whether participant was currently employed (yes/no), gender (male/female); years elapsed following initial contact with mental health services (continuous); knowledge of their diagnosis (yes/no), prior admission to hospital as a compulsory patient (yes/no), agreement with diagnosis (agree/disagree/unsure-ambivalent/know diagnosis), and current type of mental healthcare (in-patient/outpatient/treatment at home/day care/other).

### Statistical analyses

Data were analysed in STATA version 16.

Descriptive analyses assessed the distribution of socio-demographic, clinically related, independent (continuous HDI) and dependent (experienced discrimination, anticipated discrimination) variables. Mean and standard deviations were calculated for normally distributed continuous variables. For continuous variables with skewed distribution, the 25th, median, and 75th percentile were reported. Raw frequencies and percentages were reported for categorical variables to show their distribution across the dataset. Kruskal–Wallis tests were calculated to convey between-country cluster variation in experienced and anticipated discrimination scores.

Univariate and multivariable analyses involved: (i) a negative binomial regression to investigate the relationship between continuous HDI and negative experienced discrimination and (ii) a Poisson regression to investigate the relationship between HDI and anticipated discrimination. Both negative binomial regression and Poisson regression share the assumption that individual observations are independent of one another. However, individual-level observations in the dataset were clustered at the country-level, therefore participant measurements on experienced and anticipated discrimination within the same country were likely to display within-cluster correlation; Huber–White sandwich corrections to standard errors was used to account for clustering.

Univariable regression analyses were conducted to assess the unadjusted effect of country-level continuous HDI on participant reported (a) negative experienced discrimination and (b) anticipated discrimination (Model 1). Next multivariable analyses were performed by successively including potential confounders. Firstly, socio-demographic variables were entered into the regression model (Model 2) followed by clinical covariates (Model 3). All socio-demographic and clinically related covariates were maintained in the fully adjusted model irrespective of whether a confounding effect was detected. Confounding was assessed for by observing the change in the estimate of effect. *β* coefficients were exponentiated for ease of interpretation. *p*-values for univariate and multivariable analyses were ascertained from Wald tests.

Further exploratory analyses were undertaken to assess HDI in a categorical manner (medium/high/very high), to investigate whether HDI exerts a threshold effect on reported discrimination as observed in previous work exploring HDI and discrimination among people with major depressive disorder (Lasalvia *et al.*, 2015).

## Results

### Descriptive analyses

Summary statistics for the socio-demographic and clinical characteristics of the total study sample (*n* = 807) are displayed in Table 1. The age of study participants ranged from 18 to 76 years, more than half were male and unemployed. The number of years of education ranged from 0 to 38 years, with a median of 12.75 years. Similarly, the number of years since first contact with mental health services ranged from 0 to 50 years with a median of 14 years. Approximately, half of participants were being treated as an outpatient or had previously been admitted as a compulsory patient. Most participants were aware of and agreed with their diagnosis of schizophrenia.

**Table 1.** Distribution of socio-demographic and clinically related characteristics across the study sample (*n* = 807)

Socio-demographic and clinically related characteristics	*n* (%)	Mean (SD)	Missing; *n* (%)
Gender			–
Female	312 (38.66)		
Male	495 (61.34)		
Age (years)		39.40 (11.25)	–
Years of education		12.75 (3.52)	7
Currently in employment			6 (0.74)
No	565 (70.54)		
Yes	236 (29.46)		
Current type of mental healthcare			10 (1.24)
In-patient	179 (22.46)		
Out-patient	412 (51.69)		
Treatment at home	47 (5.90)		
Day-care	153 (19.20)		
Other	6 (0.75)		
Ever treated in hospital as a compulsory patient			3 (0.37)
No	372 (46.27)		
Yes	432 (53.73)		
Do you know your diagnosis			4 (0.50)
No	138 (17.19)		
Yes	665 (82.81)		
Agrees with diagnosis with Schizophrenia			53 (6.57)
Agree	473 (62.73)		
Disagree	129 (17.11)		
Unsure/ambivalent	58 (7.69)		
Don’t know diagnosis	94 (12.47)		

Using the UNDP thresholds values to illustrate the spread of data across HDIs, the distribution of countries and participants was: medium HDI (*n* = 50 participants, 2 countries); high HDI (*n* = 323 participants, 12 countries); and very high HDI (*n* = 434 participants, 15 countries). Across the participating 29 countries the median score (25th, 75th percentiles) for HDI was 0.861 (0.766, 0.896), which is indicative of very high HDI (0.8–1.0).

The frequency and percentage of participants reporting experienced discrimination and anticipated discrimination has been reported previously (Thornicroft *et al.*, 2009). In brief, there was a high level of negative discrimination experienced across the sample in interpersonal relations with friends (46.88%), family (42.45%), neighbours (29.38%) and partners (28.23%). Another key domain was employment, with disadvantages reported in keeping (30.56%) and seeking for (29.89%) a job. Anticipated discrimination was likewise common, with more than half of participants reporting anticipated discrimination across all domains assessed. Almost three-quarters (73%) felt the need to conceal their diagnosis.

Kruskal–Wallis tests showed a significant between-group variation in negative experienced discrimination scores across countries (*p* < 0.001). Conversely, anticipated discrimination scores did not display significant between-group variation across countries (*p* = 0.46).

**Figure 1.**
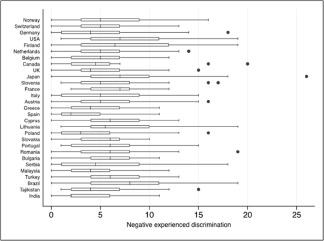
Median, 25th and 75th percentiles, interquartile ranges and outlier values for negative experienced discrimination per country, ordered by descending Human Development Index (n = 807).

The median count score for negative experienced discrimination reported by participants with schizophrenia by country ranged from 2 to 8 (see Fig. 1) with an overall median value (25th, 75th percentiles) across countries of 5 (2, 8). The median count score for anticipated discrimination by country ranged from 2 to 3 with an overall median value (25th, 75th percentiles) across countries of 3 (1, 3).

### Univariate and multivariable analyses

Table 2 displays findings from univariate and multivariable negative binomial and Poisson regression analyses modelling the association between: (a) HDI and negative experienced discrimination and (b) HDI and anticipated discrimination. For every 1 unit increase in HDI, the outcome (experienced or anticipated discrimination scores) is multiplied by the exponential of the *β* coefficient (multiplicative model). Results are reported as percentage increase or decrease in the negative experienced or anticipated discrimination scores for every 1 unit increase in HDI.

**Table 2.** Unadjusted and adjusted estimates for the association between Human Development Index and reported experienced and anticipated discrimination across the sample (*n* = 807)

	Negative experienced discrimination^a^		Anticipated discrimination^b^	
Human Development Index	Exp(*β*) (95% CI)^c^	*p* value	Exp(*β*) (95% CI)^c^	*p* value
Model 1^d^ (*n* = 807)	1.44 (0.62−3.34)	0.39	1.20 (0.86−1.66)	0.28
Model 2^e^ (*n* = 794)	1.39 (0.57−3.36)	0.47	1.22 (0.87−1.72)	0.25
Model 3^f^ (*n* = 773)	1.04 (0.45−2.45)	0.92	1.11 (0.78−1.59)	0.57

a Negative binomial regression analyses.

b Poisson regression analyses.

c Exponentiated *β* coefficients.

d Unadjusted crude association between Human Development Index and (a) negative experienced discrimination and (b) anticipated discrimination.

e Adjusted for socio-demographic covariates; gender, years of education and employment status.

f Adjusted for model 2 and clinical covariates; years since first contact with mental health services, current type of mental healthcare, ever treated in hospital as a compulsory patient and knowledge of diagnosis.

There was no evidence of a significant association between HDI and experienced or anticipated discrimination in univariate regression analyses (Model 1), or multivariable regression analyses after adjusting for socio-demographic and clinically related covariates (Model 2, Model 3).

### Exploratory analyses

There was a significant association between categorical HDI ratings and experienced discrimination whilst adjusting for socio-demographic and clinical covariates (*p* = 0.004). Experienced discrimination scores were 31% higher in high HDI countries (Brazil, Bulgaria, Cyprus, Lithuania, Malaysia, Poland, Portugal, Romania, Serbia, Slovakia, Spain, Turkey) compared to medium HDI countries (India, Tajikistan) (Exp(*β*) 1.31, 95% CI 1.10–1.56). Similarly, experienced discrimination scores in very high HDI countries (Austria, Belgium, Canada, Finland, France, Germany, Greece, Italy, Japan, Netherlands, Norway, Slovenia, Switzerland, United Kingdom, United States) were 26% higher than in medium HDI countries (Exp(*β*) 1.26, 95% CI 1.08–1.47). No countries were categorised as low HDI.

## Discussion

This study investigated whether country-level HDI is associated with individual-level experienced and anticipated discrimination in a cross-sectional sample of adults with schizophrenia across different countries across the world, via secondary analyses of data collected for the INDIGO-Schizophrenia study in 2005–2008. There was no association between HDI (as a continuous measure) and negative experienced or anticipated discrimination. However, further exploratory analyses found a significant association between HDI (as a categorical measure) and negative experienced discrimination. As the HDI of countries increased from “medium” to “high”, the frequency of negative experienced discrimination increased; however, there was a minimal difference in this frequency between “high” and “very high” HDI countries. There was no association between HDI (as a categorical measure) and anticipated discrimination.

These indicative results do mirror some other findings. In support of these findings from the exploratory analyses using data from 2005 to 2008, studies considering more recent data have likewise reported higher levels of experienced discrimination among individuals with schizophrenia in high and very high HDI nations, compared to medium HDI countries. For example, studies from very high/high HDI settings (UK (Farrelly *et al.*, 2014) and Poland (Cechnicki *et al.*, 2011)) reported higher levels of experienced discrimination compared to a study from India (Koschorke *et al.*, 2014) (medium HDI), suggesting that the pattern between HDI and discrimination suggested by these exploratory analyses is not restricted to older data, such as those considered in this secondary analysis. Moreover, in Hong Kong, up to half of participants reported negative experiences during in-patient psychiatric admissions (e.g. unnecessary use of restraint) (Lee *et al.*, 2006). This finding supports the significant association between prior compulsory hospital treatment and negative experienced discrimination observed in this study.

Considering the indicative patterns observed in the current study, one potential explanation could be the overall socioeconomic country climate. Compared to medium HDI countries, high and very high HDI contexts might foster highly competitive education and work environments with a strong focus on performance (Littlewood, 1998; Papadopoulos *et al.*, 2013). Where individuals with schizophrenia may display fluctuating performance (e.g. during a schizophrenia relapse), high expectations among teachers and employers could render these individuals vulnerable to job dismissals, inability to secure employment, and/or neglect at school. This rationale is supported by a past study (Lasalvia *et al.*, 2015) which reported significant increases in experienced discrimination with progressive HDI ratings in socioeconomic domains, such as “seeking employment”. It has also been argued that people with disabilities can be more socially engaged and economically productive in low- and middle-income countries (LMICs) as they can, for example, do lower complexity farm roles, whereas in HICs there is a higher threshold to access gainful employment (Gaebel *et al.*, 2017).

Sociocultural elements in LMICs may also play a role in the lower levels of experienced discrimination observed in the medium HDI countries in our sample. Many lower HDI settings hold collectivistic cultural values, fostering strong cohesion among families that may protect individuals from experiences of discrimination within the inner social network (Gaebel *et al.*, 2017). Conversely many high HDI societies reflect Western cultural settings with more individualistic values, where a diagnosis of schizophrenia might be viewed as a transgression of expectations of self-sufficiency. Subsequently, these views may facilitate negative attitudes and discriminatory behaviours from family and friends. Such experiences in HICs have been associated with a decrease in social capital (Webber *et al.*, 2014), which can result in poor quality of life and reduced opportunities (e.g. occupational success).

The emerging findings of this study are, however, also in contrast with some previously reported patterns. A comparable study exploring cross-national patterns of discrimination (Lasalvia *et al.*, 2015) found that anticipated discrimination among people with major depressive disorder significantly increased with higher HDI ratings. Overall, findings regarding the patterns of discrimination across different country contexts are mixed with studies also reporting that discrimination among individuals with schizophrenia is more pronounced in low and medium HDI nations. For example, in a study conducted in Kenya (Ebuenyi *et al.*, 2019) participants reported higher rates of experienced discrimination compared to findings from the INDIGO-Schizophrenia study (Thornicroft *et al.*, 2009), which included mostly high and very HDI nations. Also, mean anticipated discrimination scores in Kenya were higher than in China (Li *et al.*, 2017). Underlying factors postulated to explain this pattern include more harmful explanatory models of mental health (Girma *et al.*, 2013; Makanjuola *et al.*, 2016), harsher socioeconomic climates (Koschorke *et al.*, 2014) and adverse sociocultural factors (Gaebel *et al.*, 2017) in LMICs. Overall, it is also necessary to consider that the DISC scale assesses experienced and anticipated discrimination in different ways (more items enquire regarding former vs. the latter domain) (Brohan *et al.*, 2013; Thornicroft *et al.*, 2009), so direct comparisons between the constructs as assessed through this instrument specifically should be interpreted with caution.

These variations in global findings should be considered in view of the methodological heterogeneity often observed in stigma research. Whilst most studies used cross-sectional designs and involved facility-based samples, studies have also involved community-based samples. Additionally, methods have ranged from qualitative analyses to experimental designs. The studies have also used different instruments to assess stigma and discrimination. Moreover, whereas this study considered HDI groupings to capture variation in country contexts, previous studies have primary considered country-level differences. These differences make it challenging to draw comparisons between studies, and subsequently achieving an overall sense of patterns between country contexts and discrimination. Also, it is also necessary to consider that the DISC scale assesses experienced and anticipated discrimination in different ways (more items enquire regarding former vs. the latter domain) (Brohan *et al.*, 2013; Thornicroft *et al.*, 2009), so direct comparisons between the constructs as assessed through this instrument specifically should be interpreted with caution.

### Strengths and limitations of the study

This is the first study to examine the relationship between HDI and discrimination among people with schizophrenia. Data were collected from a large global sample across multiple countries, and the cross-national nature of this study allowed a consistent methodology to be implemented throughout study sites. The study’s focus on discrimination reflects a meaningful outcome for PWLE, and the comprehensive analysis approach utilising HDI as both a continuous and categorical measure allowed for an in-depth assessment of the association between HDI and reported discrimination.

However, no causal inferences can be made given the cross-sectional nature of the study, and its indicative exploratory findings need to be considered with caution. The analysis was limited to the covariates that were collected for the original study. There may have been additional factors not reported in the dataset that are associated with both HDI and discrimination, leaving the possibility for residual confounding. The primary INDIGO-Schizophrenia study was not designed specifically to consider differences in reported discrimination by HDI. Thus, the sample was not optimally balanced to represent the full continuum of HDI, particularly at the medium and lower ends with no low HDI countries included. Nevertheless, the indicative results of the exploratory analyses provide an initial exploration of the association between HDI and reported discrimination and highlight aspects that require verification in a more tailored sample.

It is also important to consider that these data were collected between 2005 and 2008. The 2006 HDI rating considered in these analyses is appropriately contemporaneous to the data collection, but when interpreting the results it should not be assumed that the findings are directly generalisable to the current date. It also needs to be noted that the DISC-10 measure used to assess discrimination in this study has since been superseded with DISC-12 and DISCUS for assessing experienced discrimination (Bakolis *et al.*, 2019; Brohan *et al.*, 2022), and there are now dedicated instruments to assess anticipated discrimination (Gabbidon *et al.*, 2013). These data do, however, provide a unique opportunity to consider HDI as associated with discrimination related to schizophrenia, generating important insights regarding this relationship. It is notable that no updated datasets are available to enable comparable cross-country explorations of this association. These findings should, as such, be considered an indicator or how experiences of discrimination might vary between country contexts that differ on indices, such as HDI.

### Implications

Given the dearth of cross-national stigma studies and the heterogenous evidence from prior research, there is a need for additional high-quality coordinated multi-country studies in this field to generate updated data and enable further comparisons across countries and contexts. This is needed also given the multiple anti-stigma campaigns that have been launched in recent years, and the societal changes leading to openness regarding mental health discussions in some settings. Updated multi-country datasets would enable continued explorations of differences in stigma and discrimination between different contexts, considering the potentially changed social parameters.

Use of current estimates of HDI, and updated psychometrically robust measures of discrimination validated across several country settings (e.g. using DISC-12 (Brohan *et al.*, 2013) its shortened version DISCUS (Bakolis *et al.*, 2019; Brohan *et al.*, 2022), and/or the Questionnaire on Anticipated Discrimination (Gabbidon *et al.*, 2013)), would help produce the most relevant and reliable findings.

The apparent non-linear relationship between HDI and experienced discrimination observed in this study and not reported in prior literature suggests the relationship between HDI and reported discrimination in individuals with schizophrenia deserves further investigation. Exploring this association further is warranted also given the initial, exploratory nature of the results presented in this study. Further work should aim to select a large sample representing the breadth of the HDI continuum across a wide geographic span. Greater variance in scores would allow a fuller assessment of the relationship between HDI and reported discrimination including a comparison between low and medium HDI nations which was not assessed in this sample. A larger sample size in each HDI category would permit a comparison of experienced discrimination across varying domains (e.g. interpersonal relations) stratified by HDI ratings.

Furthermore, a deeper understanding of potential sociocultural and socioeconomic indicators of discrimination may facilitate an understanding of the mechanisms that underpin cross-national differences in experienced discrimination across differing levels of HDI. Further qualitative research would contribute to an in-depth understanding of these factors from a service user perspective. Subsequent findings would further advance the adaptation of anti-stigma interventions across different contexts and cultures.

In conclusion, emerging findings from the exploratory analyses conducted for this study suggest that participants from medium-level HDI nations experienced less discrimination than those in high and very high HDI nations. Underlying sociocultural and socioeconomic differences across settings may have contributed to the apparent non-linear pattern observed. This study contributes to the global stigma literature and ongoing discussion regarding worldwide differences in discrimination among individuals with mental-ill health. Further rigorous large-scale cross-national research is required to substantiate these indicative novel findings and identify contextual factors that underpin the differences observed.

## Data Availability

The data that support the findings of this study are available on request from the senior author (graham.thornicroft@kcl.ac.uk). Consent was not sought to share data publicly.
